# Calf skinfold measurements as a diagnostic tool for lipodystrophy syndromes: a cross-sectional study

**DOI:** 10.1186/s13098-025-01934-y

**Published:** 2025-10-10

**Authors:** Grayce Ellen da Cruz Paiva Lima, Fabia Karine de Moura Lopes, Jessica Silveira Araújo, Natália Prado Boris, Virginia Oliveira Fernandes, Victor Rezende Veras, Mayara Teixeira Alexandrino Sales, Lorena Taúsz Tavares Ramos, Amanda Caboclo Flor, Ana Paula Dias Rangel Montenegro, Maria Helane Gurgel Castelo, Clarisse Mourao Melo Ponte, Júlia Lemos Lima Verde, Maria C. Foss-Freitas, Renan Magalhães Montenegro Junior

**Affiliations:** 1 Brazilian Group for the Study of Inherited and Acquired Lipodystrophies (BRAZLIPO), Fortaleza, Brazil; 2 Clinical Research Unit, Walter Cantídio University Hospital, Federal University of Ceará/EBSERH, Fortaleza, CE Brazil; 3 Department of Clinical Medicine, Federal University of Ceará, Fortaleza, CE Brazil; 4 Department of Community Health, Federal University of Ceará, Fortaleza, CE Brazil; 5 University of Fortaleza (UNIFOR), Fortaleza, CE Brazil; 6 Diagnósticos das Américas, DASA, Barueri, Brazil

**Keywords:** Lipodystrophy, Calf skinfold measurement, Body composition, Genetic diagnosis, Screening, Adipose tissue, Metabolic disorders

## Abstract

**Background:**

Lipodystrophy syndromes (LS) are characterized by reduced body fat and associated metabolic complications, such as insulin resistance and diabetes. The diagnosis of LS can be challenging in clinical settings. Skinfold measurement and body composition assessment using DEXA are commonly used for screening and diagnosis. This study aimed to identify alternative easy-to-use body composition parameters for diagnosing LS in clinical settings.

**Methods:**

This cross-sectional study included patients with genetically confirmed congenital generalized lipodystrophy (CGL) and familial partial lipodystrophy (FPL), with 62 matched healthy controls. Comprehensive data, including medical history, body composition, laboratory measurements, and imaging parameters, were collected. The receiver operating characteristic (ROC) curve was constructed using genetic test results, the gold standard for diagnosing genetic lipodystrophy, as the reference, and the sensitivity and specificity of calf skinfold measurements were measured with those of the genetic tests.

**Results:**

We included 62 patients with a mean age of 32 ± 18 (2–68) years, of whom 69.3% were women. The prevalence of diabetes mellitus (70%) and hypertriglyceridemia (83%) were also high. All body composition parameters significantly differed, except for calf skinfold measurements, which were similar between the CGL and FPL patients (*p* = 0.270). The ROC curve analysis identified a new cutoff point for calf skinfold measurement of <8 mm, with high diagnostic accuracy.

**Conclusions:**

This study identified calf skinfold measurements <8 mm as an accurate diagnostic tool for CGL and FPL. Notably, clinical accessibility to calf skinfold will favor its widespread use, standardization, and inclusion in physical examination protocols, improving lipodystrophy detection and diagnosis.

## Background

Lipodystrophy is a heterogeneous group of acquired or inherited disorders characterized by a reduction in adipose tissue. It often manifests through a complex clinical presentation that includes metabolic abnormalities, such as insulin resistance, diabetes mellitus (DM), and hypertriglyceridemia, thereby increasing the risk of adverse clinical outcomes [1–3].

Lipodystrophy syndromes are subdivided into generalized and partial forms based on the pattern of adipose tissue involvement. Familial partial lipodystrophy (FPL) typically manifests during puberty or adulthood. These cases are particularly challenging to identify, and diagnoses are often delayed due to normal variations in body composition that may confound clinicians. Currently, diagnosis is supported by medical history, body composition, and genetic criteria [4, 5].

Despite increasing accessibility to genetic testing and whole-body magnetic resonance imaging (MRI), which are considered gold standards for diagnosis, their use remains limited, especially in regions with scant financial resources [6, 7]. The availability of effective treatments for lipodystrophy necessitates early and precise diagnosis [8, 9]. Several anthropometric criteria have been proposed to support the clinical recognition of lipodystrophy. Although these indices can improve diagnostic accuracy, their complexity may limit applicability in routine settings. Due to its accessibility and ease of measurement, calf skinfold thickness may offer a simpler alternative. To address current diagnostic limitations, we aimed to identify practical and low-cost body composition parameters that could serve as complementary tools for the clinical identification of lipodystrophy.

## Methods

This cross-sectional study was conducted at the Diabetes, Dyslipidemia, and Metabolic Syndrome Outpatient Clinic of the Endocrinology and Diabetes Service at the Federal University of Ceará/Ebserh Hospital Complex (CH-UFC/Ebserh) [2, 3] from May 2022 to May 2024.

### Participants

All patients who were followed up in the outpatient lipodystrophy clinic were invited; however, only those with genetically confirmed lipodystrophy were included in the study. Molecular analysis of the following genetic panels (target sequencing) was performed: *ABCA1, AGPAT2, AKT2, APOA5, APOC2, BSCL2, CAV1, CFTR, CIDEC, CTRC, CYP27A1, GPIHBP1, LIPA, LIPE, LMF1, LMNA, LMNB2, LPL, PLIN1, POLD1, PPARG, PRSS1, PSMB8, SMPD1, SPINK1,* and *ZMPSTE24*. One patient clinically diagnosed with congenital generalized lipodystrophy (CGL), who was a biological sibling of an individual with a confirmed genetic diagnosis, was also included, even in the absence of confirmatory genetic testing.

### Control group

Data of individuals without lipodystrophy were collected from database of the Anthropometry, Body Composition, and Metabolism Laboratory (LACAM) at the Clinical Research Unit, CH-UFC/Ebserh. These individuals were matched by age, sex, and body mass index (BMI) with patients with lipodystrophy, forming two separate control groups: one for patients with CGL and the other for patients with FPL. The comparison was restricted to patients with CGL aged >8 years due to difficulties in performing skinfold and dual-energy X-ray absorptiometry (DXA) measurements in infants. For patients with FPL, the analysis was limited to those over 18 years of age, considering the natural history of FPL, wherein the clinical phenotypic expression typically begins after puberty.

### Body composition assessment

All patients were carefully examined by the same trained dietician, who recorded the following anthropometric data: body weight (kg), height (m), and BMI (kg/m^2^). Anthropometric assessments and DXA scans were conducted according to protocols that mandated fasting, abstaining from alcohol, and refraining from physical exercise for 24 h before examination [10, 11]. Women were not examined during their menstrual period. DXA assessment were conducted using GE Healthcare equipment, model Lunar Prodigy Advance™, with enCORE version 17 software.

Skinfold thickness was measured using a Lange™ skinfold caliper, calibrated. Seven standard sites (thoracic, mid-axillary, triceps, subscapular, abdominal, suprailiac, and thigh) were assessed on the right side of the body, following the Jackson and Pollock protocol [10]. In cases of limb amputation or other contraindications, measurements were taken on the contralateral side. Two measurements were obtained at each site, and the mean value was recorded.

Calf skinfold (CS) measurements was added to the protocol, due to its clinical relevance in the natural history of FPL. This measurement followed the method described by Guillín-Amarelle et al. [19], as it is not included in the original Jackson and Pollock protocol [10]. For this, the area to be assessed was first freed of clothing. Then, the patient was positioned with the knee joint flexed at a 90° angle. The examiner pinched the skin and subcutaneous fat, lifting them away from the muscle. The skinfold was measured at its greatest circumference, along the longitudinal axis on the medial line of the calf. The measurement was repeated twice to ensure accuracy, and the average of the readings was used.

### Laboratory evaluation

Blood was collected after a 12-h overnight fast, as recommended for clinical analyses. Fasting blood glucose, total cholesterol, high-density lipoprotein cholesterol (HDL-c), low-density lipoprotein cholesterol (LDL-c), and triglycerides were measured using the colorimetric method, following the manufacturer’s instructions. Glycated hemoglobin was measured with high-pressure liquid chromatography (PREMIER®-Trinity Biotech apparatus). Insulin levels were measured only in patients who did not receive insulin therapy. Homeostasis model assessment (HOMA-IR) was performed using fasting blood glucose and basal insulin levels [12]. HOMA-IR was considered elevated for values >2.5 in children and adolescents and >2.7 in adults not receiving insulin therapy [13].

### Statistical analysis

The accuracy of body composition parameters and that of genetic test results was compared using the receiver operating characteristic (ROC) curve, and the efficacy of the parameters for screening genetic lipodystrophies was determined using the area under the ROC curve (AUC). An AUC of >0.8 was considered indicative of excellent discriminative ability.

### Ethics approval

This study was approved by the Ethics Committee of the Federal University of Ceará, which ensured adherence to the ethical standards for research involving human participants. Participants or their legal guardians, in the case of minors, signed either an informed consent form or an informed assent form.

## Results

**Fig. 1:**
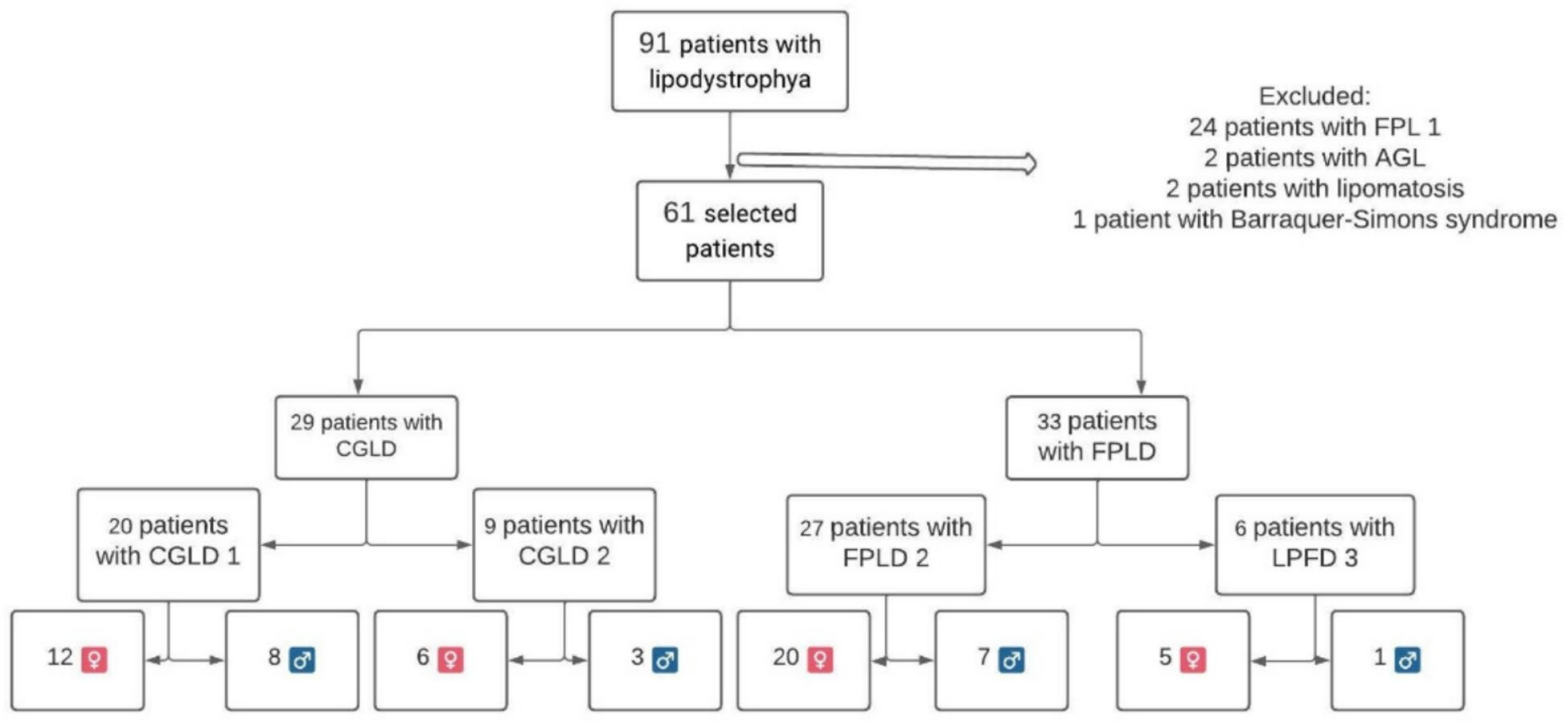
Patient selection flowchart. *CGL 1 *congenital generalized lipodystrophy type 1, *CGL 2* congenital generalized lipodystrophy type 2, *FPL 1* familial partial lipodystrophy type 1, *FPL 2* familial partial lipodystrophy type 2; *AGL* acquired generalized lipodystrophy

Of the 91 medical records of patients with lipodystrophy followed in the service, 62 were selected for participation in our reference center (Fig. 1). A total of 61 patients had a genetically confirmed diagnosis of lipodystrophy syndrome. In addition, the patient with type 2 CGL, who was the biological sister of a patient with a confirmed genetic diagnosis of CGL, met the diagnostic criteria of Patni and Garg [5].

The mean age of the patients was 32 ± 18 years. Patients with CGL had a mean age of 22 ± 12 years, whereas patients with FPL had a mean age of 41 ± 17 years (Table 1).

**Table 1:** Baseline demographic and clinical characteristics of patients with genetic lipodystrophy followed at the BRAZLIPO-CE center

Variable	All	CGL	FPL	*p*-value
Sex				0.243
Male	19 (31%)	11 (38%)	8 (24%)	
Female	43 (69%)	18 (62%)	25 (76%)	
Age* (years)	32 ± 18 31(2–68)	22 ± 12 18 (2–44)	41 ± 17 40 (6–68)	**<0.001**

*p*-values shown in bold are statistically significant

All: all patients with genetic lipodystrophies, *CGL* congenital generalized lipodystrophy, *FPL* familial partial lipodystrophy

Among the patients, 42 (70%) were diagnosed with DM, 23 (79%) of whom had CGL and 19 (61%) had FPL. Additionally, hypertriglyceridemia was observed in 50 (83%) of the 61 patients evaluated, with a prevalence of 90% in patients with CGL and 77% in those with FPL.

Regarding high-density lipoprotein cholesterol (HDL-c) levels, 51 (88%) of the 58 patients had low HDL-c levels, 26 (90%) of whom had CGL and 25 (83%) had FPL. The metabolic and biochemical parameters are presented in Table 2.

**Table 2:** Metabolic and biochemical parameters of patients with genetic lipodystrophy

Variable	N	All (n = 62)	CGL (n = 29)	FPL (n = 33)	*p*
Fasting glucose (mg/dL)	56 42	141 ± 83 101 (67–514)	151 ± 98 110 (67–514)	129 ± 63 98 (71–274)	0.611
In patients with DM	42	164 ± 87 145 (67–514)	170 ± 99 159 (67–514)	155.9 ± 64.6 144 (76–274)	0.753
Fasting insulin (uUI/mL)	36	34 ± 47 20 (0–259)	39 ± 56 22 (2–259)	24 ± 22 17 (2.1–62.2)	0.668
HOMA-IR	22	4.8 ± 4.5 3.3 (0.38–18.49)	4.3 ± 3.3 4.1 (0.38–10.24)	5.3 ± 5.4 3.2 (0–18.49)	0.974
HbA1c (%)	57	7.57 ± 2.57 6.9 (3.9–13.7)	8.11 ± 2.89 7.20 (3.9–13.7)	7.00 ± 2.10 6.1 (4.8–12)	0.090
HbA1c in patients with DM	42	8.45 ± 2.47 8 (4–13)	8.86 ± 2.69 8.3 (4.4–13.7)	7.91 ± 2.0 7.9 (4.9–12)	0.202
HDL-c (mg/dL)	57	34 ± 11 32 (12–59)	29 ± 9 27 (12–51)	39 ± 10 39 (23–59)	**<0.001**
Triglycerides (mg/dL)	58	477 ± 729 195 (40–3565)	516 ± 732 191 (72–3565)	437 ± 737 195 (40–3213)	0.474
DM	42	615 ± 819.74 258 (74–3565)	616 ± 777 298 (74–3565)	613.7 ± 871 252 (100–3213)	0.705

*p*-values shown in bold are statistically significant

All: all patients with genetic lipodystrophies, *CGL* congenital generalized lipodystrophy, *FPL* familial partial lipodystrophy, *p* *p*-value, *DM* diabetes mellitus (%)

### Body composition analysis

Body composition data obtained by anthropometry and DXA (Table 3) showed significant differences between the CGL and FPL groups, except for CS (*p* = 0.270). BMI ranged from underweight to normal weight among CGL patients, with the majority (65.5%) presenting with normal weight. Among FPL patients, BMI ranged from underweight to obesity grade 2, with varied distributions: underweight (9.4%), normal weight (47%), and overweight (25%, obesity grade 1 in 13%, and obesity grade 2 in 6.3%).

**Table 3:** Data of body composition obtained by anthropometry and dual energy X-ray absorptiometry (DXA) of patients with genetic lipodystrophy

Variable	N	All (n = 62)	CGL (n = 29)	FPL (n = 33)	*p*
Weight (kg)	61	56 ± 17 58 (18.7–104)	50 ± 15 50 (13–78.7)	61 ± 18 60 (18.7–104)	**0.015**
Height (m)	61	1.55 ± 1.16 1.59 (0.92–1.79)	1.56 ± 0.20 1.62 (0.92–1.76)	1.54 ± 0.13 1.54 (1.1–1.79)	**<0.001**
BMI (kg/m^2^)	61	22.7 ± 4.8 22.8 (14.7–35.3)	20.1 ± 2.9 20.2 (14.7 -25)	24.6 ± 5 24.7 (15.4 -35.3)	**<0.001**
SCS (mm)	50	21 ± 15 16 (5–60.5)	10 ± 4 9 (5–16)	28 ± 14 30 (7–60.5)	**<0.001**
CS (mm)	49	5.96 ± 4.12 5.00 (2–24)	4.81 ± 0.96 5 (3–6)	6.63 ± 5.03 5.5 (2–24)	0.270
TS (mm)	51	8.7 ± 4.0 8.0 (4–23.5)	6.9 ± 1.8 7.0 (4–11)	9.7 ± 4.6 8 (4–23.5)	**0.016**
BFP	55	21 ± 10 22 (6.3–42)	10 ± 2 10.3 (6.3–15)	28 ± 7 27 (12.4–42)	**<0.001**
AFP	55	21 ± 9 19 (7.7–45.1)	13 ± 3 13 (7.7–19.6)	26 ± 8 26 (12.5–45.1)	**<0.001**
LFP	55	18 ± 8 16 (6.4–36.9)	12 ± 3 13 (6.4–17.8)	22 ± 7 20 (10.4–36.9)	**<0.001**

*p*-values shown in bold are statistically significant

All: all patients with genetic lipodystrophies, *CGL* congenital generalized lipodystrophy, *FPL* familial partial lipodystrophy, *BMI* body mass index, *SCS* subscapular skinfold, *CS* calf skinfold, *TS* thigh skinfold, *BFP* body fat percentage, *AFP* arms fat percentage, *LFP* leg fat percentage, *p* p-value

Numerical data are presented as mean ± standard deviation and median (minimum–maximum). Categorical data are expressed as absolute numbers/percentages within the category

The independence chi-square test and Mann–Whitney test were used

Source: Developed by the author

### Comparative analysis of body composition, anthropometric measurements in genetic lipodystrophies

In the analysis by sex, no significant differences were observed in the CS, thigh skinfold (TS), or Leg Fat Percentage (LFP) measurements between men and women, as shown in Table 4.

**Table 4:** Comparative analysis of body composition and anthropometric measurements according to sex in patients with genetic lipodystrophy

	N	All	Men n = 17	Women n = 40	*p*
Group	57				0.173
CGL		29 (51%)	11 (65%)	18 (45%)	
FPL		28 (49%)	6 (35%)	22 (55%)	
BMI	57	22.9 ± 4.8 22.8 (14.7–35.3)	22.9 ± 5.5 22.2 (14.7–35.3)	22.9 ± 4.6 23.2 (15.1–35.2)	0.734
CS	46	5.41 ± 3.25 5.00 (2.00–23.00)	5.21 ± 2.55 4.50 (2.50–11.50)	5.50 ± 3.54 5.00 (2.00–23.00)	0.521
TS	47	8.1 ± 3.7 7.0 (4.0–23.5)	7.8 ± 4.9 6.8 (4.0–23.5)	8.2 ± 3.2 8.0 (4.0–19.0)	0.206
LFP	51	17 ± 7 16 (6–37)	16 ± 8 14 (6–34)	18 ± 7 16 (8–37)	0.189

All: all patients with genetic lipodystrophies, *BMI* body mass index, *CS* calf thickness, *TS* thigh skinfold, *LFP* leg fat percentage, *p* *p*-value

Numerical data are presented as mean ± standard deviation and median (minimum–maximum). Categorical data are expressed as absolute numbers/percentages within the category

The independence chi-square test and Mann–Whitney test were used

Fifty-two healthy individuals comprising the control group were divided into two subgroups for comparison with the CGL and FPL groups (Table 4). Four children with FPL and six with CGL were excluded from the study.

The groups were well-matched, and all body composition assessment parameters significantly differed between the groups with lipodystrophy and their respective control groups, except for the subscapular skinfold in the FPL and its control groups.

Comparative data between patients with lipodystrophy and the control group are presented in Table 5, demonstrating significant differences in the measurements obtained using anthropometry and DXA.

**Table 5:** Comparative analysis of body composition dual energy X-ray absorptiometry (DXA) and anthropometric measurements of patients with congenital generalized lipodystrophy (CGL) and familial partial lipodystrophy (FPL) and healthy subjects (control group)

Variables	CGL (n = 23)^a^	Control (n = 23)^a^	*p*-value^a^	FPL (n = 29)^b^	Control (n = 29)^b^	*p*-value^b^
Sex			>0.999			>0.999
Men	11 (38%)	11 (38%)		7 (24%)	7 (24%)	
Women	18 (62%)	18 (62%)		22 (76%)	22 (76%)	
Age (years)	24 ± 11 23 (9–44)	24 ± 11.5 23 (9–47)	0.506	41 ± 17 40 (6–68)	41 ± 17 41 (7–68)	0.904
BMI (kg/m^2^)	20.6 ± 2.8 20.6 (15–25)	21.3 ± 2.9 22.1 (15–25.7)	0.133	25.1 ± 5.2 24.7 (15.5–35.3)	25.1 ± 4.6 24.3 (16–34.9)	>0.999
SCS (mm)	10 ± 4 9 (5–16)	16 ± 7 14 (5–28)	**0.004**	28 ± 14 30 (7–61)	23 ± 10 23 (6–45)	0.153
CS (mm)	5 ± 1 5 (3–6)	18 ± 10 15 (5–40)	**<0.001**	7 ± 5 6 (2–24)	18 ± 9 16 (5–37)	**<0.001**
TS (mm)	7 ± 2 7 (4–11)	28 ± 13 25 (9–60)	**<0.001**	10 ± 5 8 (4–24)	27 ± 12 27 (7–50)	**<0.001**
LFP	12 ± 3 13 (6–18)	34 ± 9 35 (18–50)	**<0.001**	22 ± 7 20 (10–37)	34 ± 7 34 (19–48)	**<0.001**
BFP	10 ± 2 10 (6–15)	30 ± 8 31 (17–44)	**<0.001**	28 ± 7 27 (12–42)	33 ± 8 35 (11–47)	**0.004**

*p*-values shown in bold are statistically significant

*CGL* congenital generalized lipodystrophy, *FPL* familial partial lipodystrophy, *BMI* body mass index, *SCS* subscapular skinfold, *CS* calf skinfold, *TS* thigh skinfold, *LFP* Leg fat percentage, *BFP* body fat percentage

Numerical data are presented as mean ± standard deviation and median (minimum–maximum). Categorical data are expressed as absolute numbers/percentages within the category

The chi-square test for independence and Mann–Whitney test were used

^a^ comparison of patients with congenital generalized lipodystrophy (CGL) with their respective control group; ^b^ comparison of patients with familial partial lipodystrophy (FPL) with their respective control group

**Fig. 2:**
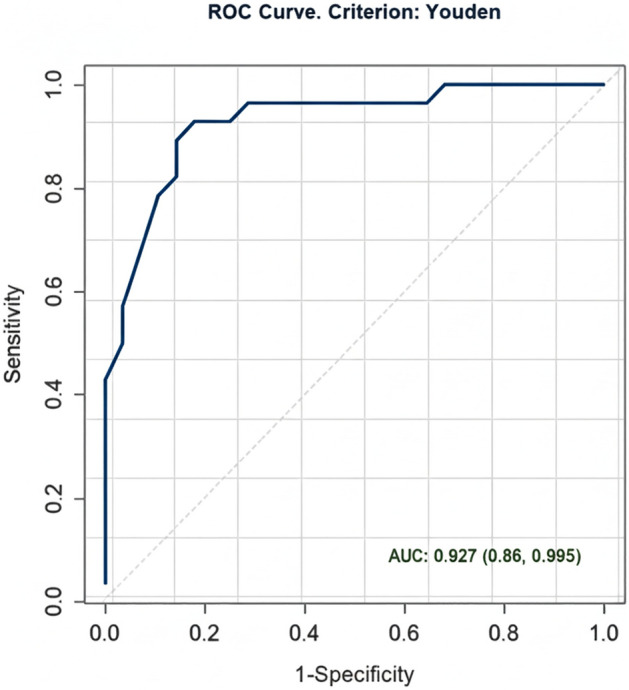
The receiver operating characteristic (ROC) curve for establishing diagnostic criteria for lipodystrophy regardless of the degree of involvement and sex, using Youden’s index. Calf skinfold: cutoff <8 mm, sensitivity 93.4%, specificity 89.5%, and area under the curve 94.7%. The positive and negative predictive values are 89.5 and 93.4%, respectively. *CS* calf skinfold. Source: Compiled by the author

The ROC curve analysis (Fig. 2) was used to evaluate the effectiveness of CS as a discriminator between patients with lipodystrophy and the general population using genetic testing as the reference test (gold standard). New cutoff points for the diagnosis of lipodystrophy were: CS of <8 mm.

### Use of body composition measurement criteria for lipodystrophy screening and clinical diagnosis

**Fig. 3:**
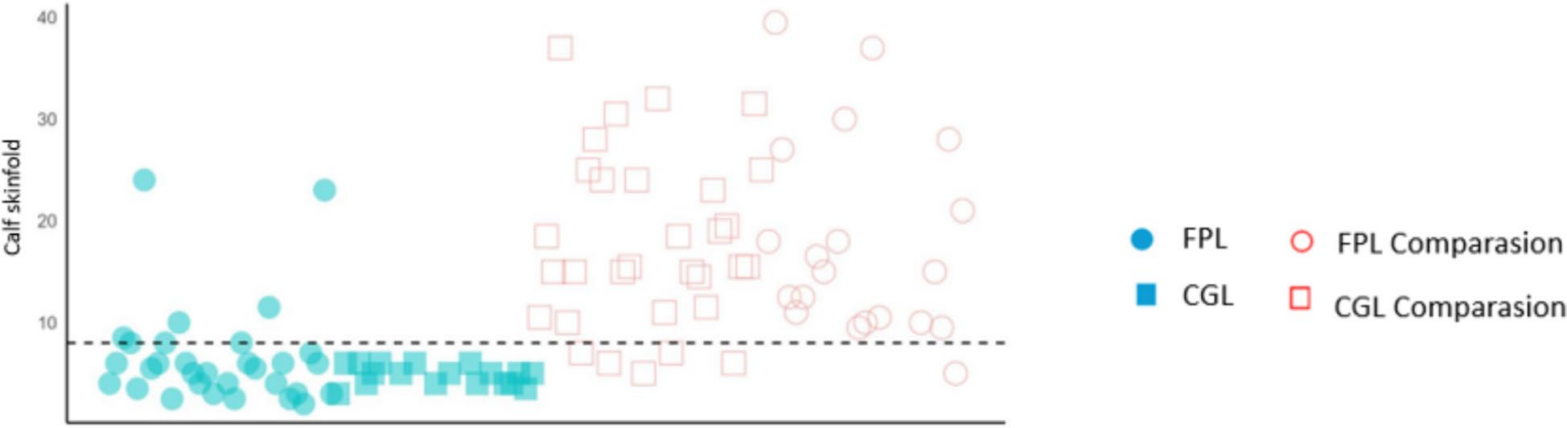
Scatterplots of calf skinfold measurement in patients with lipodystrophy and control groups. Scatterplot of values obtained from calf skinfold measurements; the dashed line represents a cutoff point of 8 mm. *Blue circle*, familial partial lipodystrophy (FPL); *blue square*, congenital generalized lipodystrophy (CGL); *pink circle*, FPL control group; *pink square*, CGL control group

After establishing the previously mentioned cutoff point, a scatter plot including all measured values in the lipodystrophy and control groups was constructed to examine the applicability of this criteria (Fig. 3).

All individuals with CGL and the majority with FPL presented measurements of <8 mm for CS. However, in three adult FPL patients, these measurements exceeded these limits: a 24-year-old woman (CS: 23 mm); a 68-year-old man (CS: 10 mm), who presented with a milder metabolic phenotype with DM that developed only at the age of 65 years; and a 66-year-old man (CS: 11 mm), who presented an unfavorable clinical picture, characterized by various micro and macrovascular complications.

### ROC curve analysis by sex and lipodystrophy type

Subsequently, further analyses were conducted to explore the sex specific impact on the cutoff values in specific regions. This approach involved generating separate ROC curves for each sex, facilitating a more detailed examination of variations. The findings from these analyses, including the cutoff values, along with their corresponding sensitivity, specificity, and AUC data, are summarized in Table 6. Similarly, considering the distinct pathophysiologies among the different types of genetic lipodystrophy, cutoff points were explored according to the extent of adipose tissue involvement (Table 6).

**Table 6:** Comparison between cutoff points for screening and clinical diagnosis of genetic lipodystrophy according to sex and degree of adipose tissue involvement

Group	Sensitiviy	95% CI sensitivity	Specificity	95% CI specificity	AUC	95% CI AUC
All ♂♀	0.935	1–1	0.896	0.583–0.979	0.947	0.903–0.992
All ♀	0.969	1–1	0.971	0.309–1	0.975	0.936–1
All ♂	0.857	0.786–1	0.714	0.262–1	0.880	0.753–1
CGL ♂♀	1	1–1	0.95	0.85–1.000	0.978	0.933–1
CGL ♂	1	1–1	0.875	0.625–1.000	0.961	0.878–1
CGL ♀	1	1–1	1.000	1.000–1.000	1.000	1–
FPL ♂♀	0.893	1–1	0.857	0.595–0.964	0.927	0.86–0.995
FPL ♂	0.667	0.333–1	0.500	0.167–1	0.764	0.469–1
FPL ♀	0.955	1–1	0.955	0.292–1.000	0.962	0.905–1

All: all patients with genetic lipodystrophies, *CGL* congenital generalized lipodystrophy, *FPL* familial partial lipodystrophy, *S* sensitivity, *E* specificity, AUC area under the curve, mm: millimeters, *♂* male individuals, *♀* Female individuals, *CI* confidence interval

Source: Developed by the author

Based on a thorough analysis of sensitivity, specificity, and Youden’s index at different cutoff points on the ROC curve for each evaluated parameter, an increase in sensitivity was observed, with a slight decrease in specificity among the male group compared with the female group. These findings support the decision to standardize the cutoff points for both sexes and lipodystrophy subtypes. Therefore, considering the values obtained when analyzing the group as a whole, as presented in Table 6, a single cutoff value of 8 mm was established to encompass all the variations observed across different subgroups and measurements.

## Discussion

This cross-sectional study evaluated the body composition parameters in patients with genetic lipodystrophies and presented a new useful anthropometric parameter for the screening and clinical diagnosis of lipodystrophy. Early detection of this disease is crucial as it may reduce premature morbidity and mortality [14].

Anthropometric and body composition evaluation using DXA and skinfold measurements are rapid, practical, and economically accessible methods for the diagnosis of lipodystrophies compared with gold-standard methods such as MRI and genetic testing [15, 16]. Previous studies have highlighted some anthropometric parameters as useful for the diagnosis of lipodystrophy, including TS [17, 18], Köb index [19], fat mass ratio [20], LFP [21], and leg fat mass and total fat mass ratio [22]. However, there are no universally accepted anthropometric or body composition parameters, particularly for partial lipodystrophies [23]. In this study, significant differences between the CGL and FPL groups were found in all anthropometric and body composition parameters, except for CS. Considering genetic testing as the gold standard for the diagnosis of genetic lipodystrophies, ROC curve analysis defined a cutoff point for CS of <8 mm for both CGL and FPL, achieving excellent accuracy in both sexes.

This finding should be interpreted in the context of existing anthropometric indices. The Köb index, which is based on the ratio between subscapular and calf skinfold thickness, has previously been suggested as a tool for identifying FPL, with a cutoff >3.477 indicating a high probability of the condition [24]. However, such composite indices require multiple measurements and calculations, which may reduce their practicality in clinical settings. In contrast, calf skinfold thickness alone demonstrated comparable diagnostic performance in our sample and offers a simpler, faster, and more discreet method, potentially increasing its utility in daily clinical practice, especially in resource-limited environments. These thresholds were not pre-specified but were derived from the study data using ROC curve analysis.

Currently, the most commonly used clinical criterion for identifying lipodystrophy is TS of <22 mm in women and <10 mm in men, based on the observation that >90% of people without lipodystrophy have measurements above these values [17, 18]. A recent study on Brazilian women with FPL identified a cutoff point of 20 mm for TS. Despite the small and heterogeneous sample, which included FPL type 1 diagnosed without a specific genetic confirmatory test, this finding suggests that the cutoff point for the Brazilian female population may be lower [25].

In 2020, Vasandani et al. suggested that an FPL below the 1st percentile, according to the National Health and Nutrition Examination Survey (NHANES) curves, could be used to diagnose partial lipodystrophy [21]. However, the practical use of this criterion is challenging, particularly owing to variations in FLP values among different age groups [1, 26].

Examining the distribution of CS measurements in all patients with lipodystrophy, we observed that a CS criterion of <8 mm was present in all patients with CGL and almost all patients with FPL.

In the two cases with negative results based on CS measurements, individual characteristics are believed to have influenced the findings. In the case of the 24-year-old woman, the negative result may be explained by the natural history of FPL, where clinical phenotypic expression usually begins after puberty. In contrast, the 65-year-old man, classified as a false negative in the clinical evaluation of CS but confirmed positive through genetic testing via cascade screening, exhibited a milder metabolic phenotype. This suggests variability in gene expression associated with lipodystrophy. These cases emphasize the importance of a detailed clinical history and a thorough understanding of the disease's natural progression, providing valuable insights into its variability and diagnostic challenges.

Physiological variations in adipose tissue distribution between men and women suggest different cutoff points for diagnosing lipodystrophy based on sex [17, 18]. In this study, patients with lipodystrophy were categorized into male and female groups to compare body composition and anthropometric measurements. The analysis showed that the CS, TS, and LFP measurements did not differ significantly between the sexes. To reduce the influence of potential confounding variables, patients were stratified by sex, and comparisons of anthropometric and body composition parameters accounted for differences in BMI and age.

When the impact of lipodystrophy type was evaluated, the cutoff points were lower in the CGL group than in the FPL group. However, standardizing a single value for these cutoff points also resulted in only a slight reduction in specificity and an increase in sensitivity.

Standardizing cutoff points without distinguishing between sex or lipodystrophy type simplifies the use of these tests by healthcare professionals, enhancing diagnostic sensitivity and reducing the risk of false negatives. Thus, body composition assessment is a reliable tool for the screening and diagnosis of lipodystrophy. The adjusted cutoff points were validated in all analyses, except for LFP in men with FPL, where the small number of patients may have affected the results. These findings highlight the relevance of these new thresholds as effective diagnostic methods for lipodystrophy.

These results emphasize the use of CS measurements for the effective screening of patients with lipodystrophy. It is particularly notable for its simplicity and convenience, requiring less physical exposure to the patient, thus maintaining privacy during consultation.

It is also important to note that lipodystrophies may be found in various contexts, including hypercortisolism, menopause-associated lipodystrophy, and certain medication use [18, 27]. Thus, in addition to anthropometric and body composition assessments, a comprehensive medical evaluation is mandatory to differentiate them from genetic forms. A major clinical challenge involves the differential diagnosis between FPL) and conditions such as severe metabolic syndrome and central obesity, which often share overlapping phenotypic and metabolic characteristics [28, 29]. Similarly, in suspected cases of CGL, it is critical to distinguish affected individuals from those with naturally low body fat, including athletes and individuals with low body fat percentage. In this context, a comprehensive diagnostic approach is essential to enhance diagnostic accuracy and guide appropriate clinical management. This should incorporate a detailed clinical history—particularly the identification of family members with similar phenotypic features and parental consanguinity—alongside anthropometric assessments, such as calf skinfold thickness measurement, and complementary laboratory and imaging studies.

A limitation of the current study was the small number of patients included, due to the rarity of this disease. In particular, the number of male patients with FPL was very limited, which may have affected the sensitivity and specificity of the calf skinfold measurement in this subgroup. However, it is important to emphasize that this is a robust Brazilian series of patients with both forms of genetic lipodystrophy [2, 3]. The cross-sectional design of the study allows for the analysis of important associations between anthropometric parameters and lipodystrophy, providing a foundation for future investigations, including longitudinal studies.

## Conclusion

This study evaluated the body composition and anthropometric parameters in patients with genetic lipodystrophy and identified calf skinfold measurements <8 mm as an accurate diagnostic tool for CGL and FPL. Standardizing this cutoff point, regardless of sex or lipodystrophy type, resulted in a slight reduction in specificity but improved sensitivity. Notably, the practical clinical accessibility of calf skinfold measurement promotes its widespread use, standardization, and inclusion in physical examination protocols, improving the detection and diagnosis of lipodystrophy.

## Data Availability

No datasets were generated or analysed during the current study.

## Abbreviations

DM: Diabetes mellitus
FPL: Familial partial lipodystrophy
MRI: Magnetic resonance imaging
CGL: Congenital generalized lipodystrophy
BMI: Body mass index
DXA: Dual-energy X-ray absorptiometry
CS: Calf skinfold
HOMA-IR: Homeostasis model assessment
ROC: Receiver operating characteristic
AUC: Area under the ROC curve
HDL-c: High-density lipoprotein cholesterol
TS: Thigh skinfold
FLP: Fat leg percentage
SCS: Subscapular skinfold
BFP: Body fat percentage
AFP: Arms fat percentage
S: Sensitivity
E: Specificity
AUC: Area under the curve
